# Vacuole protein sorting 18 (*Vps18*) suppresses epithelial growth factor receptor (EGFR) expression and lung tumorigenesis

**DOI:** 10.1016/j.jbc.2025.110447

**Published:** 2025-07-02

**Authors:** Wuhou Dai, Shujing Zhang, Shunfei Yan, Hao Zhang, Sha Zou, Tonglu Yu, Wufan Tao, Hengning Ke, Xingrong Du

**Affiliations:** 1 The Institute of Developmental Biology and Molecular Medicine, Fudan University, Shanghai, P.R. China; 2 Hubei AIDS Clinical Training Center, Department of Infectious Disease, Zhongnan Hospital, Wuhan University, Wuhan, P.R. China; 3 Shanghai Key Laboratory of Metabolic Remodeling and Health, Institute of Metabolism and Integrative Biology, Drug Clinical Trial Center, Shanghai Xuhui Central Hospital, Zhongshan-Xuhui Hospital, Fudan University, Shanghai, P.R. China

**Keywords:** Vps18, lung cancer, EGFR, K-Ras mouse, tumorigenesis

## Abstract

Lung cancer remains the leading cause of cancer-related deaths worldwide. While lysosomal degradative function is critical for cellular homeostasis, its role in lung cancer pathogenesis remains poorly understood. Here, we identify *Vps18* as an important tumor suppressor in murine lung tumorigenesis. Genetic ablation of *Vps18* accelerates lung tumorigenesis in LSL-*K-Ras* mice, accompanied by enhanced tumor cell proliferation. Mechanistically, *Vps18* deficiency elevates EGFR protein levels and activates the ERK-MAPK signaling pathway in lung tumors. Strikingly, expression of dominant-negative EGFR (*dnEGFR*) partially suppresses the tumor-promoting effects of *Vps18* loss. Our findings unveil a novel *Vps18*-EGFR-ERK axis in lung cancer and may inform the development of targeted therapeutic strategies.

Lung cancer remains the leading cause of cancer-related deaths worldwide, yet the molecular mechanisms driving its development are not fully understood. Intracellular vesicle trafficking plays a critical role in cellular processing, such as membrane recycling, biomaterial degradation, nutrient sensing, exocytosis, and cell survival (1). This process is mediated by three key protein families: Rab GTPases, tether proteins, and soluble N-ethylmaleimide-sensitive fusion protein attachment protein receptors (SNAREs) (1). Rab GTPases initiate the fusion process through a GDP-to-GTP switch, recruiting tethers and other effector proteins (2). Tether proteins then establish the initial contact between vesicles, bringing them into proximity for SNARE-mediated fusion (3). Growing evidence implicates lysosome-related processes in carcinogenesis and metastasis (4, 5). Several tethering complex subunits have been linked to cancer progression. For instance, *Vps33 B* deletion induces hepatocarcinoma (HCC) in mice (6), while *Vps8* mutation in *Drosophila* leads to melanotic tumors (7). These findings highlight tethering complex subunits as potential therapeutic targets.

The class C Vps complex, composed of VPS11, VPS16, VPS18 and VPS33, regulates late endosome and lysosome-related vesicle transport (1). As a core subunit, the VPS18 protein is essential for proper vesicle trafficking. In yeast,*Vps18* deletion disrupts vacuole structure and causes autophagosome and late endosome accumulation (8). Similarly, *Caenorhabditis elegans* lacking *Vps18* exhibits defective endosome–lysosome biogenesis and impaired degradation of cellular debris (9). In *Drosophila*, *dor* (the *Vps18* homolog) mutants display loss of retinal pigmentation, enlarged multivesicular bodies (10, 11), and enhanced tumor growth and metastasis (12). Zebrafish *Vps18* deficiency leads to hepatomegaly, skin hypopigmentation, and reduced retinal melanosomes (13). In mammalian cells, disruption of *Vps18* function impairs autophagosome maturation and early endosome fusion (14, 15). Neural-specific deletion of *Vps18* in mice causes severer neurodegeneration and aberrant neuronal migration due to blocked lysosomal trafficking (16). Additionally, *VPS18* contributes to cancer drug resistance, and its knockdown suppresses xenograft tumor growth (17). However, the mechanisms by which *VPS18* influences tumor development remain unclear.

Here we identify *Vps18* as a novel tumor suppressor in lung cancer. We show that *Vps18* loss accelerates lung tumorigenesis in LSL-*K-Ras* mice, accompanied by enhanced tumor cell proliferation. Mechanistically, *Vps18* deficiency increases EGFR protein levels and activates the EGFR-MAPK signaling pathway in mouse lung tumors. Importantly, this tumor-promoting phenotype is partially rescued by dnEGFR expression in mouse lung tumors. Our findings uncover a novel *Vps18*-EGFR-ERK axis in lung cancer and shed light on the design of new therapeutic strategies.

## Results

### *Vps18* deficiency promotes lung tumorigenesis in mice

To investigate the function of the *Vps18* gene in lung tumorigenesis, *Vps18*^*+/F*^ *K-Ras* mice were generated by crossing *Vps18*^*F/F*^ mice with *K-Ras* mice, and *Vps18*^*F/F*^ *K-Ras* mice were generated by self-breeding of *Vps18*^*+/F*^ *K-Ras* mice. We first assessed the effect of *Vps18* deficiency on lung tumorigenesis in *Vps18*^*+/F*^ *K-Ras*, *Vps18*^*F/F*^ *K-Ras*, and control *K-Ras* mice infected with Ad-*Cre*, followed by a microPET/CT scan (see Materials and methods).

**Figure 1:**
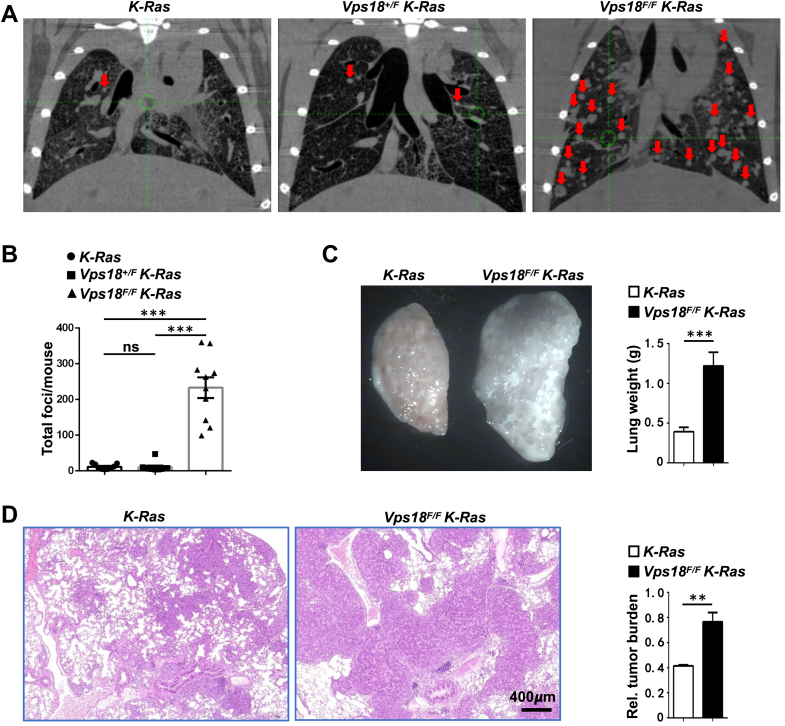
***Vps18* deficiency promotes mouse lung tumorigenesis**. *A-B*, Representative microPET/CT images of mouse lung and statistical analyses of tumor numbers in the lung from mice 2 months after Ad-*Cre* intranasal inhalation. *Red arrows* in (*A*) indicate tumor foci detected by microPET/CT in the mouse lung. Each symbol in (*B*) represents an individual mouse. *C*, representative images of whole-mount single lung specimens of *Vps18*^*F/F*^*K-Ras* and control *K-Ras* mice 8 weeks after Ad-*Cre* infection and statistical analyses of whole lung weight. *D*, representative histologic images of H&E-stained lung sections from mice 8 weeks after tracheal instillation of Ad-*Cre* and statistical analyses of tumor burden. At least 4 mice in (*C*) and (*D*) for each genotype. Values in (*B*), (*C*), and (*D*) represent the means ± SD. ∗∗, *p* < 0.01 or ∗∗∗, *p* < 0.001; ns, not significant.

The micro PET/CT results revealed no significant difference in lung tumor numbers between *Vps18*^*+/F*^ *K-Ras* mice and *K-Ras* mice (hereafter, *K-Ras* mice were used for the control unless specified). However, *Vps18*^*F/F*^ *K-Ras* mice exhibited a dramatic increase in the total number of lung tumors (Fig. 1, *A* and *B*). To confirm these findings, we also evaluated lung tumorigenesis in *Vps18*^*F/F*^ *K-Ras* mice infected with Lenti-*Cre*. Consistent with the previous results, *Vps18*^*F/F*^ *K-Ras* mice showed a significant increase in total lung weight (Fig. 1*C*) and tumor burden (Fig. 1*D*) compared with controls. These findings suggest that *Vps18* deficiency strongly promotes lung tumorigenesis in *K-Ras* mice.

### *Vps18* deficiency enhances cell proliferation in mouse lung tumors

**Figure 2:**
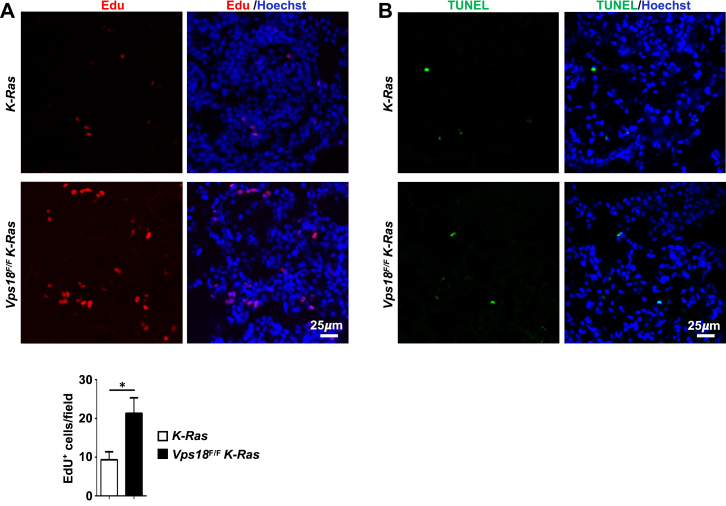
***Vps18* deficiency enhances the proliferation of lung tumor cells in mice.***A*, representative fluorescent image of mouse lung sections stained for EdU-positive cells (*red*) in lung tumors and the statistical analyses of EdU-positive cells. *B*, representative fluorescent images of mouse lung sections stained for TUNEL-positive cells (*green*) in lung tumors. The mouse genotypes are as indicated. Values in (*A*) represent the means ± SD. ∗, *p* < 0.05.

To elucidate the cellular mechanisms by which *Vps18* deficiency promotes lung tumorigenesis, we assessed cell proliferation and apoptosis in lung tumors of *Vps18*^*F/F*^ *K-Ras* and control *K-Ras* mice. Our *in vivo* EdU-pulse labeling experiments revealed significantly increased cell proliferation in the tumors from *Vps18*^*F/F*^ *K-Ras* mice compared to the controls (Fig. 2*A*). In contrast, the TUNEL assay showed no significant difference in apoptosis between these two groups (Fig. 2*B*). Together, these data suggest that *Vps18* deficiency promotes mouse lung tumorigenesis by enhancing cell proliferation.

### *Vps18* deficiency leads to upregulation of EGFR

Normal endocytic function ensures proper degradation or recycling of membrane-associated receptors and other signaling molecules. Upon ligand binding, EGFR is internalized from the plasma membrane to the early endosome, where it is sorted for either recycling to the cell surface or degradation through the lysosome pathway (18). Defects in trafficking can result in EGFR accumulation in cells, leading to enhanced signaling and cancer progression (19).

**Figure 3:**
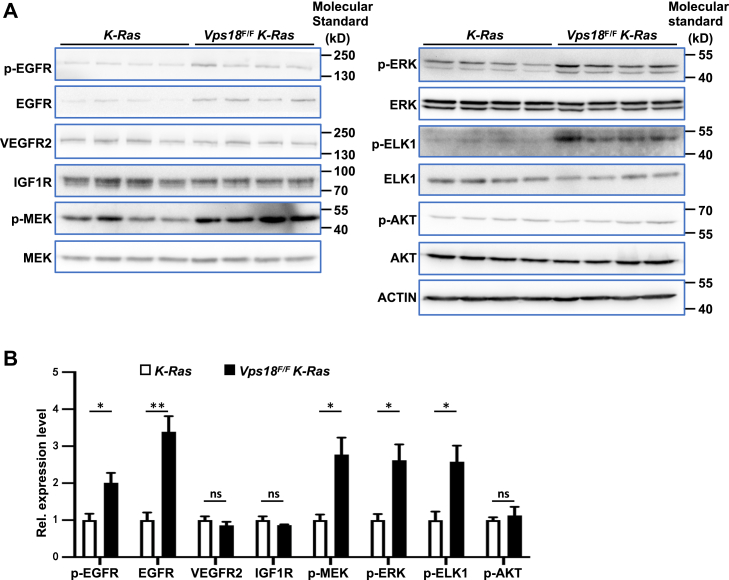
***Vps18* deficiency leads to upregulation of EGFR signaling in mouse lung tumors**. *A*, Western blotting for p-EGFR, EGFR, VEGFR2, IGF1R, p-MEK, MEK, p-ERK, ERK, p-ELK1, ELK1, p-AKT, and AKT in lung tumors from *Vps18*^*F/F*^*K-Ras* and control mice. β-Actin was used as a loading control. *B*, statistical analyses of protein levels in (*A*). Values in (*B*) represent the means ± SD. ∗, *p* < 0.05; ∗∗, *p* < 0.01; ns, not significant.

*Vps18* is a key player in endosome-lysosome trafficking (8, 9) and its deficiency blocks endosomal/lysosomal trafficking and protein degradation in multiple species (1, 8, 9, 12, 20), resulting in an accumulation of membrane proteins, such as integrins, in neuronal cells (16). We hypothesized that the enhanced EGFR signaling may contribute to the accelerated development of lung tumors in *Vps18*^*F/F*^ *K-Ras* mice. To test this hypothesis, we examined EGFR and other receptor tyrosine kinases (RTKs) in lung tumors from *Vps18*^*F/F*^ *K-Ras* and control mice by western blot. Total EGFR and phospho-EGFR levels were markedly increased in tumors from *Vps18*^*F/F*^ *K-Ras* mice compared with controls, whereas other RTKs such as IGFR and VEGFR remained unaffected. Additionally, downstream EGFR signaling components (MEK, ERK, and ELK1) were activated in tumors from *Vps18*^*F/F*^ *K-Ras* mice, though their protein level were not changed (Fig. 3 *A* and *B*). These results suggest that *Vps18* deficiency promotes lung tumorigenesis by disrupting endo-lysosome trafficking, leading to EGFR accumulation and sustained oncogenic signaling in lung tumor cells.

### Expression of dnEGFR alleviates the lung tumor-promoting phenotype in *Vps18*^*F/F*^*K-Ras* mice

**Figure 4:**
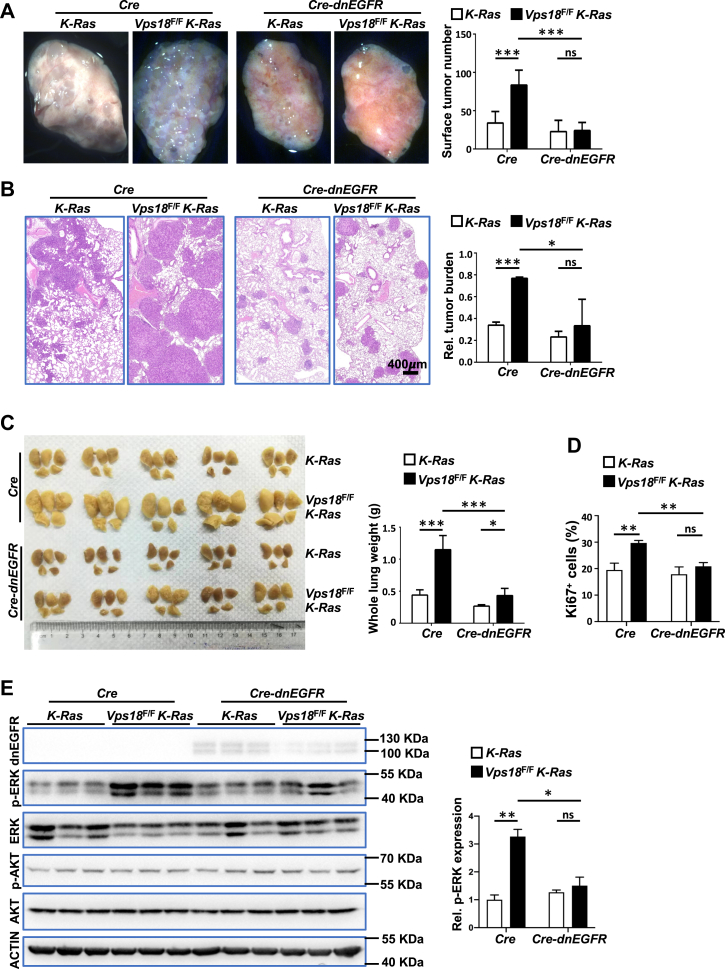
***dnEGFR* attenuates *Vps18* deficiency-induced lung tumor promotion in mice**. *A*, representative images of whole-mount single lung specimens from mice (*left*) 12 weeks after lentivirus infection, along with statistical analyses of tumor count on the surface of whole lung (*right*). *B*, illustrative histologic images of H&E-stained sections of mouse lungs in (*A*) (*left*), accompanied by statistical analyses of tumor burden (*right*). *C*, photographs of whole mount lungs from mice 12 weeks after lentivirus infection (*left*) and statistical analyses of lung weights (*right*). *D*, statistical analyses of Ki67-positive cells in tumors of immunochemistry-stained lung sections from (*B*). *E*, representative western blot of myc-tagged dnEGFR and EGFR down-stream targets (total ERK/p-ERK and total AKT/p-AKT) (*left*), and statistical analysis of p-ERK levels (*right*) in lung tumors from the mice in (*A*). ACTIN was used for loading control. The mouse genotypes and virus treatments are as indicated. At least 3 mice for each condition. Values in *A*-*E* represent the means ± SD. ∗, *p* < 0.05; ∗∗, *p* < 0.01; ∗∗∗, *p* < 0.001; ns, not significant.

To determine whether *Vps18* deficiency promotes tumor development by enhancing EGFR signaling, we investigated whether expressing dominant negative *EGFR* (*dnEGFR)* could suppress the lung tumor-promoting phenotype caused by *Vps18* loss. We constructed a Lenti-*Cre-*P2A-*dnEGFR* virus co-expressing *Cre* recombinase and *dnEGFR* and administered it *via* tracheal instillation into *Vps18*^*F/F*^ *K-Ras* and control *K-Ras* mice. Lung tumor development was assessed 12 weeks later. Western blot confirmed dnEGFR expression in tumors from mice infected with Lenti-*Cre*-P2A*-dnEGFR* virus (Fig. 4*E*).

While *Vps18*^F/F^ *K-Ras* mice infected with the Lenti-*Cre* virus exhibited significantly increased lung tumor development compared to controls, expression of *dnEGFR* abolished this effect. No significant differences were observed in either lung surface tumor number (Fig. 4*A*) or total tumor burden (Fig. 4*B*) between *Vps18*^F/F^ *K-Ras* and control mice infected with Lenti-*Cre*-P2A*-dnEGFR* virus. These results indicate that *dnEGFR* effectively suppresses the lung tumor-promoting phenotype caused by *Vps18* deficiency. Consistent with the above tumor phenotype results, *dnEGFR* expression in *Vps18*-deficient tumors normalized cell proliferation and EGFR-MAPK signaling to control levels (Figs. 4*D* & S1*A*). Together, these findings demonstrate that *Vps18* deficiency promotes lung tumor development in mice by elevating EGFR protein levels within tumor cells.

### Patients with low *VPS18* expression have a poor prognosis for lung cancer

**Figure 5:**
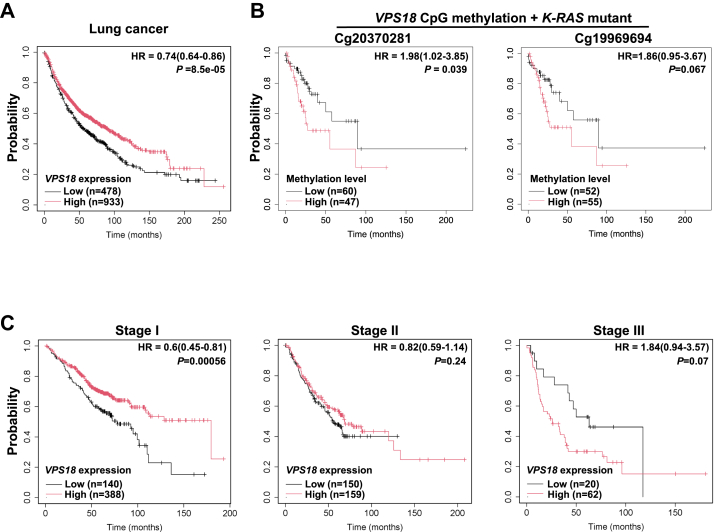
***VPS18* expression and prognosis for human lung cancer**. *A*, Kaplan–Meier survival analysis of 1411 lung cancer patients with *low* and *highVPS18* mRNA level in lung cancer samples (original data from TCGA). *B*, Kaplan–Meier survival analysis of *K-RAS* mutant lung cancer patients with *low* and high *VPS18* methylation level with two different CpG probes in lung cancer samples. *C*, Kaplan–Meier survival analysis of patients with Stage I ∼ III lung cancer having *low* and *highVPS18* mRNA level in lung cancer samples.

To investigate the clinical relevance of *VPS18* in human lung cancer, we first compared its mRNA expression between normal lung tissue (n = 75) and primary tumors (n = 560) using TCGA data. *VPS18* expression was significantly lower in tumors than in normal tissue (Fig. S3). We then assessed the prognostic impact of *VPS18* by analyzing gene expression data from 1411 patients with TCGA lung cancer. Kaplan–Meier survival analysis revealed that low *VPS18* expression was associated with significantly worse prognosis compared to high expression, with a hazard ratio (HR = 0.67, *p*-value = 8.5 × 10^-5^) (Fig. 5*A*)

To explore the potential mechanisms underlying reduced *VPS18* expression in human lung tumors, we analyzed *VPS18* copy number variation (CNV) and promoter methylation in TCGA lung cancer samples. GISTIC2 analysis of 1017 samples showed no complete copy loss, with *VPS18* CNV raging from ∼ 1 to 3. Since promoter hypermethylation often suppresses gene expression in tumors, we evaluated methylation levels of *VPS18* gene in lung tumors using data of the Illumina HumanMethylation450 BeadChip (450k) arrays. Two CpG probes (cg19969694 and cg20370281) near *VPS18* exon 1 exhibited elevated methylation among 830 primary tumors (mean β-values: 0.49773 and 0.59342, respectively). Given that homozygous (but not heterozygous) *Vps18* KO enhances lung tumor development in *K-Ras* mice, we further assessed the impact of *VPS18* copy number loss and promoter hypermethylation on overall survival in patients with lung cancer harboring *K-RAS* mutations. High methylation at cg19969694 (β ≥ 0.58810) trended toward worse survival (*p* = 0.067), while high methylation at cg20370281 (β ≥ 0.78359) significantly correlated with poorer outcomes (*p* = 0.039) (Fig. 5*B*).

Additionally, stratification by clinical stage revealed that low *VPS18* expression was associated with poorer prognosis only in patients with stage I (n = 528) (Fig. 5*C*, left). No significant association was observed in stage II (n = 309) (Fig. 5*C*, middle), while stage III (n = 82) showed a non-significant inverse trend (limited by sample size) (Fig. 5*C*, right), suggesting that other factors may dominate prognosis in advanced disease.

Together, these results suggest that *VPS18* downregulation contributes to lung cancer progression and serves as a poor prognostic marker, particularly in early-stage lung cancer.

## Discussion

Lung cancer is still the leading cause of cancer deaths worldwide, partly due to an incomplete understanding of its molecular mechanisms. In this study, we demonstrate that both lung tumor number and burden are significantly increased in *Vps18*^*F/F*^ *K-Ras* mice compared to controls. This effect is accompanied by enhanced tumor cell proliferation, elevated EGFR protein levels, and activation of EGFR-MAPK signaling. Notably, the tumor-promoting effects of *Vps18* deficiency were partially rescued by expressing *dnEGFR* in mouse lung tumors. Furthermore, lung cancer patients with low *VPS18* expression exhibit poor prognosis. Together, these findings provide the first *in vivo* evidence that *Vps18* functions as a tumor suppressor in mammalian lung cancer.

The role of *Vps18* in cancer is complex and context-dependent. Our data show that *Vps18* deficiency promotes lung tumorigenesis in *K-Ras* mice, and low *VPS18* expression correlates with worse clinical outcomes in patients with lung cancer. Consistent with these findings, Segala *et al*. found that patients with low *VPS18* expression had a poor prognosis for breast cancer (21). Supporting this tumor-suppressive role, studies in *Drosophila* and zebrafish have demonstrated that loss of *Vps18* homologs leads to increased tumor growth, metastasis, and cancer-associated phenotypes such as hepatomegaly (13).

However, the relationship between *VPS18* expression and cancer prognosis varies across tumor types. For example, Segala *et al*. and Niu *et al*. reported separately that high levels of *VPS18* mRNA are associated with relapse in gastric cancer (21) or worse prognosis in gastric cancer, bladder and liver carcinoma, and lung adenocarcinoma (17), contradicting our observation in lung cancer.

These discrepancies may arise from methodological differences, including variations in cancer stages analyzed. In our study, stratification by clinical stage revealed that low *VPS18* expression was significantly associated with worse prognosis only in patients with Stage I (Fig. 5*C*, left), with no significant association in stage II (Fig. 5*C*, middle) and a non-significant inverse trend in Stage III (Fig. 5*C*, right). Additionally, our analysis included all lung cancer patients from the TCGA database (n = 1411; 478 low vs. 933 high *VPS18*), whereas Niu *et al*. focused exclusively on patients with lung adenocarcinomas (n = 504; 367 low vs. 137 high *VPS18*). These differences in patient cohorts and cancer subtypes may explain the conflicting conclusions.

The dual roles of *VPS18* in cancer biology—acting as either a tumor suppressor or a promoter—likely depend on tissue context, cancer type, and molecular environment. This complexity highlights the need for caution when interpreting gene function in cancer and underscores the importance of context in evaluating prognostic and therapeutic implications. For lung cancer, further research is needed to clarify the conditions under which *VPS18* expression influences outcomes, which may lead to novel therapeutic strategies and prognostic biomarkers.

## Experimental procedures

### Animals

*Vps18*^*F/F*^ mice were generated by C. Peng *et al*. (16), and the LSL-*K-Ras*^*G12D*^ mice (thereafter called *K-Ras* mice) carrying a LSL*-K-Ras*^*G12D*^ allele in the C57BL/6J genetic background were previously described (22). All mice were maintained on 12/12-h light/dark cycles. Experiments were conducted with consent from the Animal Care and Use Committee of the Institute of Developmental Biology and Molecular Medicine at Fudan University, Shanghai, China.

### Lung tumor induction, enumeration, and tumor burden analysis

Lung tumors were induced by the method described previously (23). Briefly, 8-week-old *Vps18*^*+/F*^ *K-Ras*, *Vps18*^*F/F*^ *K-Ras*, and control *K-Ras* mice were infected with 5 × 10^7^plaque-forming units (PFU) of adenovirus expressing *Cre* (Ad-*Cre*) by intranasal inhalation or tracheal instillation. Two months after Ad-*Cre* infection, the mice were euthanized, and a microPET/CT scan was performed on a SIEMENS Inveon scanner (Siemens). Images were analyzed using Inveon Research Workplace (Siemens Healthcare). Tumor foci (≥0.3 mm diameter spherical regions) on mouse lungs were counted based on microPET/CT images. Eight weeks after Ad-*Cre* infection, mouse lungs were retrieved, and tumors on the mouse lung surface were counted under a dissection microscope. For tumor burden analysis, lungs were perfused through the trachea with 4% paraformaldehyde and fixed overnight, followed by standard procedures for paraffin sections and H&E staining. 6 randomly selected lung sections/mice were scanned for at least three mice each genotype. Total lung area occupied by tumor was measured and tumor burden was calculated as previously described by Lei *et al*. (24). For *dnEGFR* rescue experiments, 10^6^ transforming units (TU) of lentivirus expressing *Cre* (Lenti-*Cre* or Lenti-*Cre-*2A*-dnEGFR*) was administered by tracheal instillation. Twelve weeks after lentivirus infection, mouse lung tumor development was evaluated in the same manner as described above for adenovirus-infected mice.

### Plasmids

For plasmid pFUW-*Cre*-2A-*dnEGFR*, P2A and *dnEGFR* fragment (25) was generated by PCR from mouse cDNA and in-frame cloned at the end of the *Cre* gene in pFUW-*Cre*. For plko.1-scramble, plko.1-sh*VPS18* plasmid, the oligos cloned into the Age I and EcoR I sites of plko.1 were listed below: plko.1-scramble: CAACAAGATGAAGAGCACCAA, plko.1-sh*VPS18*: CTCTACCGAGAAACCAAGGAA.

### Cell culture, virus preparation, and infection

HEK293T cells were cultured in high-glucose DMEM with 10% FBS. The preparation of Ad-*Cre* and Lenti-*Cre* was described by Lei *et al*. (24). Recombinant lentivirus, Lenti-*Cre-dnEGFR*, was generated by co-transfecting plasmid pFUW-*Cre*-2A-*dnEGFR* and packaging plasmids (pCMV-VSV-G, pRSV-Rev, and pMDLg/pRRE) into HEK293T cells, and culture supernatant was harvested 48 h later. Then, lentiviruses were concentrated as described previously (26). TU of Lenti-*Cre* and Lenti-*Cre-dnEGFR* was determined by infecting HEK293T cells carrying LSL-EGFP transgenes following the methods published (24). As for lentiviruses for knockdown experiments, culture supernatant was harvested 48 h later and stored at −80 °C or directly used to infect the human lung cancer cell line (A549 cell line, a gift from Westlake University cell bank) followed by puromycine selection (2 ug/ml).

### Western blot

Proteins were extracted from mouse tumors using RIPA buffer with freshly added 1 mM PMSF and 1x proteinase inhibitor (Roche Applied Science). Then, proteins were resolved on SDS-PAGE followed by western blotting with antibodies against the following proteins: EGFR (ab52894), VEGFR2 (ab315238), IGF1R (ab182408) from abcam company; VPS18 (10901-1-AP) from proteintech; Phospho-EGF Receptor (3777), p44/42 MAPK (Erk1/2) (4695), Phospho-p44/42 MAPK (Erk1/2) (4370), MEK1/2 (8727), Phospho-MEK1/2 (9154), Akt (pan) (4691), Phospho-Akt (4060), Elk-1(51,398), Phospho-Elk-1 (9186), Ki67 (9129), Myc-Tag (2040) from Cell Signaling Technology; β-Actin(A3854) from Sigma-Aldrich. The protein bands were detected with an ECL Western Blotting Analysis System, and images were obtained with a Tanon-5200. The density of protein bands was determined by Image J.

### TUNEL assay, EdU incorporation assay, and immunochemistry

TUNEL assay was performed following the manufacturer’s instructions (11684795910, Roche). For the EdU (5-ethynyl-2′-deoxyuridine) assay, mice were injected intraperitoneally with 50 mg/kg EdU 8 weeks after virus infection. Two days after EdU injection, lung tissues were retrieved and subjected to frozen section, followed by EdU staining with a kit (C10310, RiboBio) according to the manufacturer’s instructions. At least 5 lung sections/mice were used for EdU staining for three mice of each genotype. EdU-positive cells on four randomly selected fields/sections were calculated for statistical analysis. Ki67 immunohistochemistry (IHC) was performed with formalin-fixed paraffin-embedded (FFPE) sections using a two-step method as described (27) with a kit (M&R HRP/DAB Detection IHC Kit, HC301–03, Vazyme) according to the manufacturer’s instructions. Percentages of Ki67-positive cells in four randomly selected fields/sections for three mice of each genotype were calculated for statistical analysis.

### Prognosis analysis of patients with lung cancer

Kaplan–Meier survival analyses were performed using the KMplot.com online tool (http://kmplot.com). We specifically utilized the Affymetrix gene chip (microarray) data for lung cancer from the TCGA and GEO datasets. Patients were stratified into high and low expression groups based on the auto-selected best cutoff for the gene of interest (*VPS18*), and survival differences were evaluated using the log-rank test. Overall survival (OS) was used as the primary endpoint. Gene-level GISTIC2 copy number data from The Cancer Genome Atlas (TCGA) lung cancer cohort and the COSMIC (Catalogue of Somatic Mutations in Cancer) database were used to analyze copy number variation (CNV) of the *VPS18* gene. Methylation data for *VPS18* were obtained from the Illumina HumanMethylation450 BeadChip (450k) arrays in the TCGA lung cancer dataset.

### Statistical analysis

Unpaired Student’s *t* test was used. A value of ∗, *p* < 0.05; ∗∗, *p* < 0.01; or ∗∗∗, *p* < 0.001 denoted statistical significance.

## Data availability

All data are contained within the manuscript.

## Supporting information

This article contains supporting information.

## Conflict of interest

The authors declare that they have no conflicts of interest with the contents of this article.
