# Supplementary material for: Vacuole protein sorting 18 (Vps18) suppresses epithelial growth factor receptor (EGFR) expression and lung tumorigenesis
Source: J Biol Chem. 2025 Jul 2;301(8):110447. doi: 10.1016/j.jbc.2025.110447 (PMC12329117; doi:10.1016/j.jbc.2025.110447)
Supplement: Supplementary Data 1 [file mmc1.docx]

**Supplemental Figure S1** Analysis of proliferation and apoptosis of lung tumor cells.

**A**. Representative immunochemistry images of mouse lung sections stained with Ki67 in lung tumors. **B**. Representative fluorescent images of mouse lung sections stained for TUNEL-positive cells (red) in lung tumors. The mouse genotypes and virus treatments are as indicated.

**Supplemental Figure S2** Knock-down of *VPS18* expression in the A549 cell does not affect EGFR expression or proliferation.

**A-B.** qRT-PCR analysis (**A**) and growth curve measurements (**B**) of *VPS18* knock-down (sh*VPS18*) and control A549 cells. **C**. Western blot analysis of VPS18, EGFR and ERK/p-ERK proteins in sh*VPS18* and control A549 cells (left) and their quantification (right). ACTIN serves as a loading control.

**Supplemental Figure S3** Analysis of *VPS18* mRNA level in normal lung and lung tumor tissues**.**

Statistical analyses of *VPS18* mRNA level between normal solid tissue and primary lung tumor. Each symbol represents ﻿one individual.
