# Supplementary figures and images for: Vacuole protein sorting 18 (Vps18) suppresses epithelial growth factor receptor (EGFR) expression and lung tumorigenesis
Source: J Biol Chem. 2025 Jul 2;301(8):110447. doi: 10.1016/j.jbc.2025.110447 (PMC12329117; doi:10.1016/j.jbc.2025.110447)

# Supplementary Figure S1

**A**

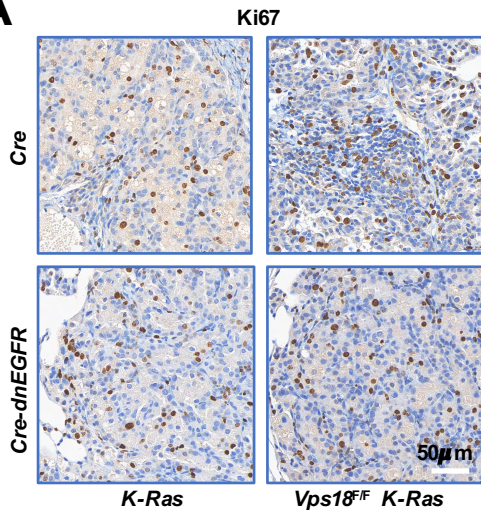

**B**

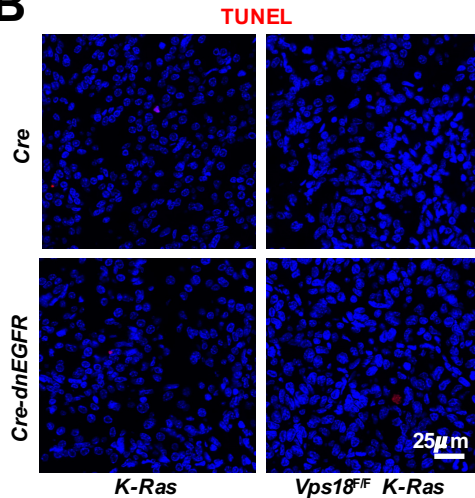

Supplement: Figure S1 [file mmc2.pdf]

# Supplementary Figure S2

**A**

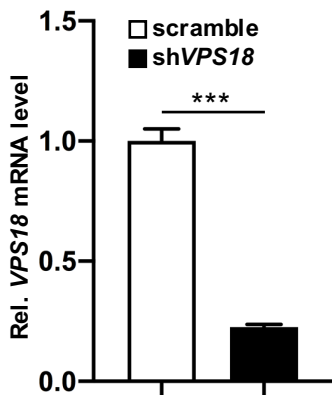

**B**

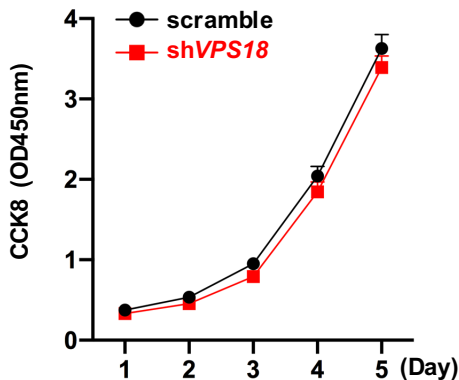

**C**

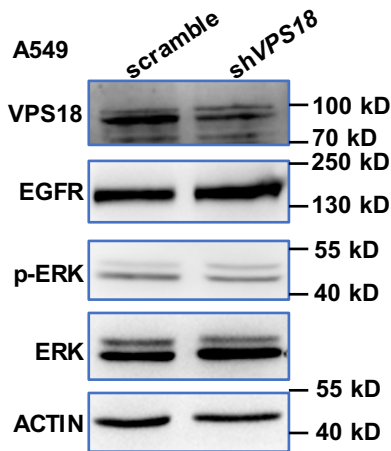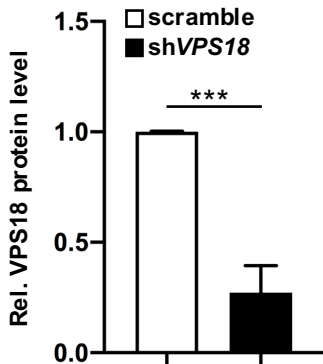

Supplement: Figure S2 [file mmc3.pdf]

# Supplementary Figure S3

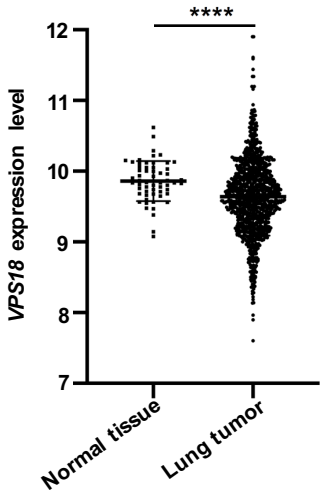

Supplement: Figure S3 [file mmc4.pdf]
